# Recurrent Superficial Thrombophlebitis Secondary to Migrated Silicone: A Diagnostic Challenge

**DOI:** 10.1016/j.jaccas.2026.107957

**Published:** 2026-04-22

**Authors:** Raghad Abuhalimeh, Musa'ab Moh'd Alhmouz, Lina Abuhalimeh, Lucy Adams, Nour Bader, Miguel Bravo, Yousef Abuhalimeh, Batool Jamal Abuhalimeh

**Affiliations:** a University of Jordan, Amman, Jordan; b Heart, Vascular & Thoracic Institute, Cleveland Clinic Abu Dhabi, Abu Dhabi, UAE; c Department of Biology Science Division, New York University, Abu Dhabi, UAE; d Cleveland Clinic Abu Dhabi, Abu Dhabi, UAE; e Aqaba Medical Sciences University, Aqaba, Jordan

**Keywords:** lower extremity swelling, silicone migration, superficial venous thrombophlebitis

## Abstract

**Background:**

Lower extremity swelling is a common clinical presentation with a broad and overlapping differential diagnosis. Although recurrent cellulitis may be commonly seen in such cases, the coexistence of migratory superficial venous thrombophlebitis and chronic edema is uncommon, particularly in the absence of varicose veins. Delayed complications of cosmetic silicone fillers are increasingly recognized; however, vascular manifestations remain rare.

**Case Summary:**

We report a 39-year-old woman with a 3-year history of recurrent bilateral lower extremity edema, cellulitis, and migratory superficial thrombophlebitis. Extensive evaluation excluded infectious, lymphatic, cardiac, renal, hepatic, malignant, autoimmune, and hypercoagulable causes. Duplex ultrasonography revealed “snowstorm” echogenicity, and magnetic resonance imaging confirmed migrated silicone deposits in the lower extremities. Surgical removal resulted in complete symptom resolution.

**Take-Home Messages:**

Delayed silicone migration can induce chronic inflammatory and vascular complications, posing a diagnostic challenge and mimicking recurrent cellulitis. This case highlights the multifactorial complexity of chronic lower extremity swelling and the importance of multidisciplinary evaluation.

## Visual Summary

**Figure:**
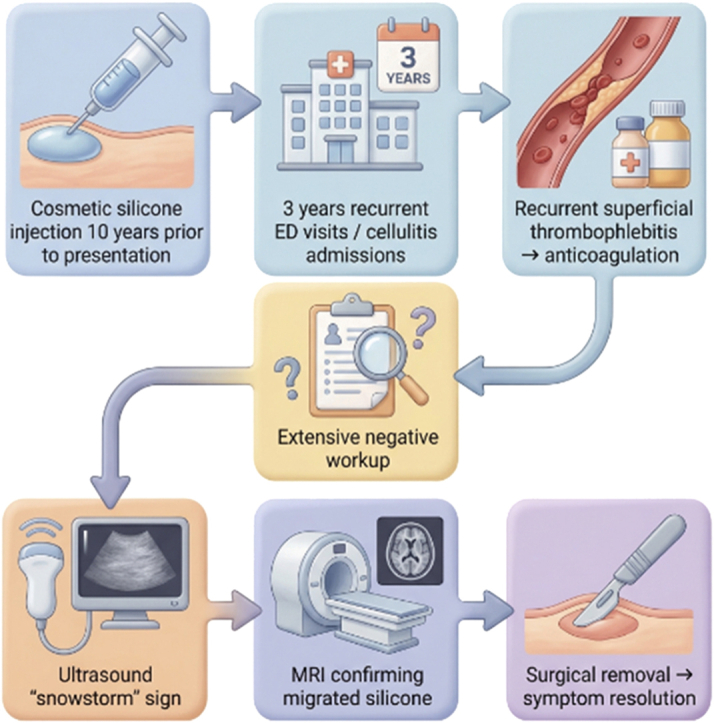

**Visual Summary:**
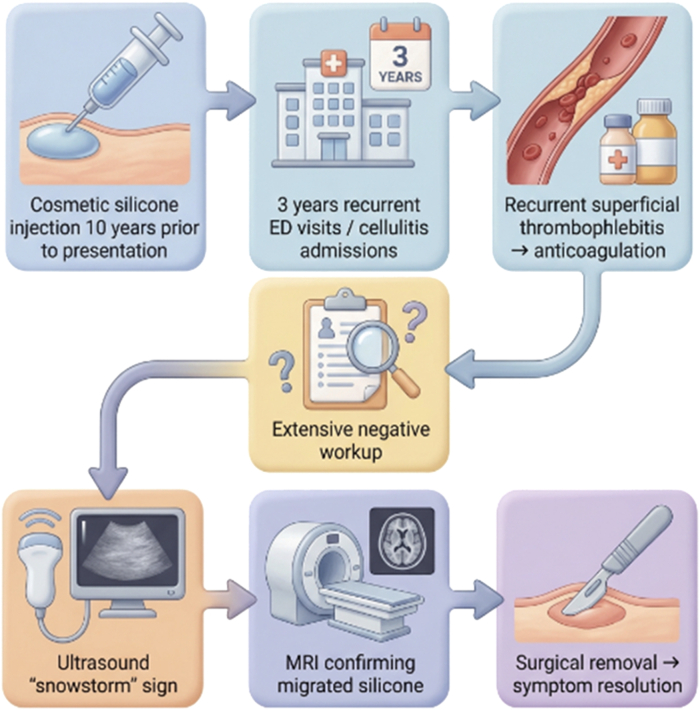
Clinical Timeline Causing Recurrent Lower Extremity Inflammation in the Patient Image created with the assistance of artificial intelligence–generated illustration tools under the direction of the authors. All clinical content, interpretation, and final editing were performed and verified by the authors. ED = emergency department; MRI = magnetic resonance imaging.

## History of Presentation

A 39-year-old woman was referred to the vascular medicine outpatient clinic for evaluation of chronic bilateral lower extremity swelling, recurrent lower extremity cellulitis, and recurrent migratory superficial venous thrombophlebitis. Review of her medical records revealed 11 emergency department visits over 3 years, including 5 hospital admissions for presumed bilateral lower extremity cellulitis requiring intravenous antibiotics. On examination, the bilateral lower extremities demonstrated diffuse, nonpitting edema extending from the thighs to the calves. There were areas of mild tenderness over the posterior thighs and popliteal fossae. The overlying skin showed mild erythema without warmth, fluctuance, or purulent discharge at the time of evaluation. No active ulceration or skin breakdown was noted.

**Take-Home Messages**

• Migratory superficial venous thrombophlebitis is a rare, delayed complication of cosmetic silicone migration and should be considered when routine etiologies are excluded.
• Multimodality imaging, including duplex ultrasonography demonstrating a characteristic “snowstorm” pattern and MRI confirmation, is essential for diagnosis.
• Early multidisciplinary evaluation and surgical removal of migrated silicone can be curative and prevent prolonged morbidity and repeated hospitalizations.

## Past Medical History

• Recurrent lower extremity cellulitis
• Migratory superficial venous thrombophlebitis
• No significant systemic comorbidities identified
• Remote history of bilateral gluteal silicone filler injections (10 years prior)

## Differential Diagnosis

• Recurrent cellulitis
• Chronic venous insufficiency
• Lymphedema
• Lipoedema
• Hypercoagulable state
• Autoimmune or inflammatory disorders
• Malignancy-associated thrombosis
• Foreign body reaction (silicone migration)

## Investigations

Prior duplex ultrasonography demonstrated noncompressible lower extremity superficial veins with scattered small oval and rounded echogenic foci, consistent with superficial venous thrombophlebitis. Extensive laboratory evaluation including hypercoagulable testing, age-appropriate malignancy screening, and autoimmune/rheumatologic work-up was unremarkable. The patient was initially managed with fondaparinux; however, persistent recurrent migratory superficial venous thrombophlebitis despite therapy necessitated transition to rivaroxaban, which was continued long-term owing to ongoing recurrence and persistence of symptoms despite anticoagulation.

**Figure 1:**
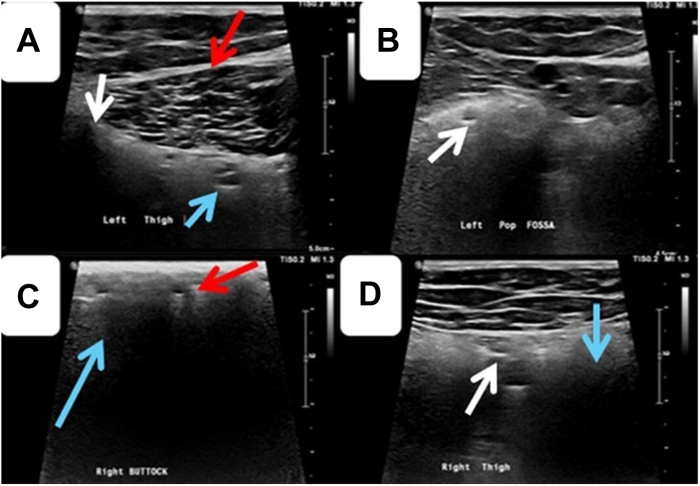
B-Mode Ultrasound of the Bilateral Lower Extremities Demonstrating Migrated Injectable Material (A) B-mode ultrasound of the left thigh demonstrates preserved muscle belly architecture (red arrow) with involvement of the deep fascial plane, where heterogeneous hyperechoic material containing multiple cystic spaces is seen (blue arrow). Prominent posterior acoustic shadowing surrounds the muscle and deep fascia, consistent with foreign material deposition (white arrow). (B) Ultrasound of the left popliteal fossa shows cysts (white arrow). (C) B-mode ultrasound of the right buttock reveals ill-defined hyperechoic soft tissue changes with multiple small cystic areas (red arrow) and a characteristic “snowstorm” artifact with posterior shadowing (blue arrow), suggestive of migrated injectable material. (D) Ultrasound of the right thigh demonstrates more extensive cystic changes (white arrow), marked hyperechogenicity, loss of normal tissue planes, and prominent posterior acoustic shadowing (blue arrow), consistent with migrated injectable material associated with a chronic inflammatory granulomatous reaction.

**Figure 2:**
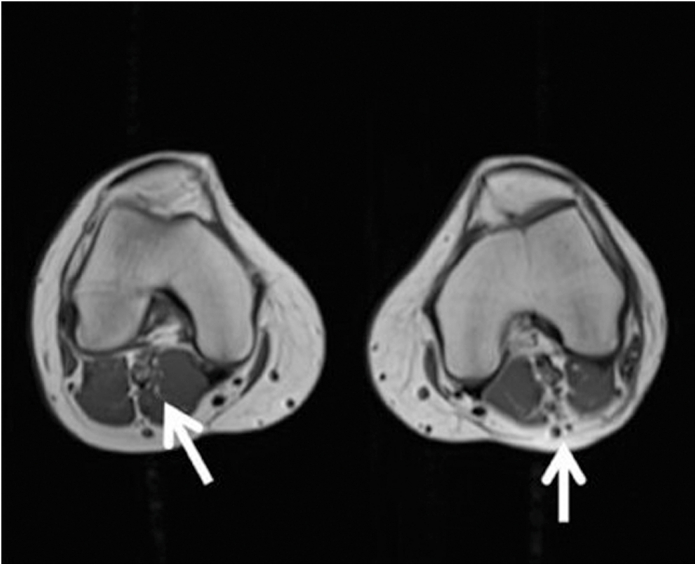
Axial Magnetic Resonance Imaging of the Bilateral Knees Demonstrating Migrated Injectable Material Axial magnetic resonance imaging demonstrate multiple rounded signal voids and cystic-appearing foci within the subcutaneous soft tissues surrounding the distal femur and popliteal regions bilaterally (white arrows). These findings are associated with distortion of normal soft tissue architecture and are consistent with migration of injectable material, likely silicone, with surrounding chronic inflammatory and granulomatous reaction. The underlying osseous structures and deep musculature appear relatively preserved.

Given refractory bilateral edema and persistent symptoms, further evaluation was pursued. Lymphoscintigraphy excluded primary or secondary lymphedema. Transthoracic echocardiography revealed normal cardiac structure and function. Renal, hepatic, and thyroid function tests, as well as serum albumin, were within normal limits. Venous ultrasonography demonstrated extensive regions of “snowstorm” echogenicity interspersed with anechoic cystic spaces throughout the buttocks, posterior thighs, and posterolateral calf, corresponding to areas previously presumed to represent cellulitis (Figure 1). These abnormalities involved both subdermal tissues and deeper myofascial planes, including the popliteal fossae and adjacent vascular beds (Figure 1). Subsequent magnetic resonance imaging of the lower extremities confirmed multiple foci of silicone within the gluteal regions and migrated silicone deposits in the adipose tissue of the bilateral popliteal fossae (Figure 2).

## Management

Upon further detailed history taking in vascular medicine clinic, the patient reported undergoing bilateral gluteal silicone filler injections approximately 10 years prior, without immediate complications. She reported that this information had not been disclosed during earlier hospital admissions because the injections had been performed abroad many years earlier and she initially did not consider them medically relevant to her symptoms. She denied any intervening trauma, surgery, or injury since. Plastic surgery consultation was obtained, and surgical excision of the migrated silicone was recommended given its suspected role in recurrent lower extremity inflammation, swelling, and cellulitis-like and superficial venous thrombophlebitis episodes.

## Outcome and Follow-Up

The patient underwent successful surgical removal, and at follow-up, she reported complete resolution of her lower extremity symptoms.

## Discussion

Bilateral lower extremity swelling represents a common yet diagnostically complex clinical presentation, encompassing a broad spectrum of cardiac, vascular, lymphatic, inflammatory, systemic, and malignant etiologies. In our case, recurrent bilateral lower extremity edema accompanied by erythema, pain, and migratory superficial thrombophlebitis prompted an extensive diagnostic evaluation. The differential diagnosis included primary and secondary lymphedema, chronic venous insufficiency, systemic inflammatory and autoimmune disorders (including vasculitis and connective tissue diseases), occult malignancy in the context of migratory thrombophlebitis (Trousseau syndrome), inherited and acquired hypercoagulable states, and cardiac, renal, hepatic, or endocrine causes of edema. Comprehensive investigations excluded infectious, lymphatic, cardiac, hepatic, renal, malignant, autoimmune, and thrombophilia etiologies, redirecting clinical focus toward structural abnormalities and foreign body–related pathology.

Injectable silicone fillers, particularly when administered in large volumes or outside regulated medical settings, have been associated with late adverse sequelae, including granulomatous inflammation, migration, vascular compromise, and persistent inflammatory responses.^1^, ^2^, ^3^ Migration may occur years after injection and is facilitated by gravity, tissue planes, and lymphatic or vascular dissemination. Prior literature has documented delayed lower extremity complications after silicone exposure, including chronic edema, inflammation, and recurrent cellulitis-like presentations.^1^, ^2^, ^3^ A case report described progressive secondary lymphedema developing over 5 years after large-volume silicone injections to the hips and buttocks, accompanied by painful granulomas at both injection and distant sites.^4^ In a cross-sectional study of 77 individuals with massive silicone injections, lymphatic or subcutaneous migration occurred in 59%, inflammation in 50%, and varicose veins in 39%, with manifestations often emerging years after initial exposure.^1^ Silicone migration from ruptured breast implants presenting as bilateral lower-leg nodules further highlights the potential for distant dissemination.^5^ Although superficial thrombophlebitis is a rare, delayed complication of silicone implant, the established association between silicone-induced inflammation, venous insufficiency, and chronic edema provides a biologically plausible substrate for superficial venous thrombosis.

Migrated silicone can precipitate thrombophlebitis through multiple mechanisms, including direct mechanical irritation of vessel walls, activation of the coagulation cascade, and inflammatory responses triggered by silicone particles in the bloodstream.^6^ When silicone migrates from its original injection or implant site, it may enter the vascular system and induce endothelial injury, with subsequent activation of coagulation pathways playing a central role in the pathogenesis of silicone-related vascular complications.^6^ Silicone particles in circulation can trigger inflammatory cascades similar to those observed in fat embolism syndrome, sharing common pathogenic mechanisms such as coagulation activation and endothelial disruption.^6^

The inflammatory response to silicone is mediated in part by macrophage activation, which drives persistent production of inflammatory mediators and oxidant metabolites long after the initial exposure.^7^ This sustained inflammatory response contributes to vessel wall damage and promotes thrombosis formation. Chronic silicone diffusion into the circulation has been documented after massive injections, with silicone-containing vacuoles detectable within monocytes and associated dermatologic manifestations, including varicose vein development.^1^ Continuous exposure of vessel walls to silicone establishes a persistent foreign-body reaction that further predisposes to vascular injury.^1^^,^^6^^,^^7^

Silicone may also interact with collagen and other extracellular matrix components, compromising vessel wall integrity and creating a prothrombotic environment.^8^ Together, direct endothelial injury, chronic inflammation, and activation of the coagulation cascade create conditions conducive to the development of superficial thrombophlebitis in vessels exposed to migrated silicone. Clinically, this mechanism helps explain the recurrent migratory superficial thrombophlebitis observed in our patient, which persisted despite anticoagulation and was refractory to conventional therapies.

The diagnostic complexity in cases of silicone migration extends beyond clinical evaluation to imaging interpretation. Multimodality imaging is therefore critical for establishing the diagnosis. In patients with a history of lower extremity silicone injections who present with findings suggestive of superficial thrombophlebitis, ultrasonography is essential both to confirm the presence of superficial vein thrombosis and to assess for silicone migration, as it has demonstrated high specificity for detecting silicone within soft tissues.^9^ Duplex ultrasonography characteristically reveals a “snowstorm” echogenic pattern, a recognized sonographic signature of free silicone, while magnetic resonance imaging can delineate the extent of silicone dissemination.^9^

Although delayed lower extremity complications of silicone migration, such as swelling and recurrent cellulitis, have been reported, this case represents a rare instance of gluteal silicone filler migration presenting with vascular manifestations and recurrent inflammatory episodes. To our knowledge, there are no prior reports documenting silicone migration in the lower extremities manifesting specifically as migratory superficial thrombophlebitis. Nonetheless, the literature does describe related vascular and inflammatory sequelae that share clinical features with superficial thrombophlebitis, highlighting the need for a multidisciplinary approach in evaluation and management.

## Conclusions

Delayed migration of cosmetic silicone fillers represents a rare but important cause of chronic lower extremity inflammation and vascular complications. This case illustrates how silicone-induced inflammatory reactions can mimic cellulitis and precipitate recurrent superficial thrombophlebitis, leading to repeated hospitalizations and prolonged diagnostic uncertainty. Comprehensive evaluation, including detailed patient history and multimodality imaging, is essential when conventional etiologies are excluded. Early recognition and multidisciplinary collaboration are critical to establish the diagnosis and guide definitive management. Surgical removal of migrated silicone can be curative, emphasizing the importance of considering foreign body–related pathology in patients with unexplained recurrent lower extremity edema and inflammatory manifestations.

## Funding Support and Author Disclosures

The authors have reported that they have no relationships relevant to the contents of this paper to disclose.
